# PhoB Regulates the Survival of *Bacteroides fragilis* in Peritoneal Abscesses

**DOI:** 10.1371/journal.pone.0053829

**Published:** 2013-01-15

**Authors:** Shin Wakimoto, Haruyuki Nakayama-Imaohji, Minoru Ichimura, Hidetoshi Morita, Hideki Hirakawa, Tetsuya Hayashi, Koji Yasutomo, Tomomi Kuwahara

**Affiliations:** 1 Department of Immunology and Parasitology, Institute of Health Biosciences, The University of Tokushima Graduate School, Tokushima, Japan; 2 Department of Microbiology, Faculty of Medicine, Kagawa University, Kagawa, Japan; 3 School of Veterinary Medicine, Azabu University, Kanagawa, Japan; 4 Kazusa DNA Research Institute, Chiba, Japan; 5 Frontier Science Research Center, University of Miyazaki, Miyazaki, Japan; University Medical Center Utrecht, The Netherlands

## Abstract

In response to phosphate limitation, bacteria employ the Pho regulon, a specific regulatory network for phosphate acquisition. The two-component signal transduction system of PhoRB plays a crucial role in the induction of Pho regulon genes, leading to the adaptation to phosphate starvation. Herein, we identified the PhoRB system in *Bacteroides fragilis*, a commensal gut bacterium, and evaluated its role in gut colonization and survival in peritoneal abscesses. BF1575 and BF1576 encoded PhoR (sensor histidine kinase) and PhoB (response regulator) in the sequenced *B. fragilis* strain YCH46, respectively. Transcriptome analysis revealed that deletion of *phoB* affected the expression of 585 genes (more than 4-fold change) in *B. fragilis*, which included genes for stress response (chaperons and heat shock proteins), virulence (capsular polysaccharide biosynthesis) and phosphate metabolism. Deletion of *phoB* reduced the ability of the bacterium to persist in peritoneal abscesses induced by an intra-abdominal challenge of *B. fragilis*. Furthermore, PhoB was necessary for survival of this anaerobe in peritoneal abscesses but not for *in vitro* growth in rich media or in intestinal colonization. These results indicate that PhoB plays an important role in the survival of *B. fragilis* under stressful extraintestinal conditions.

## Introduction

Environmental sensing and adaptive response are essential activities for all organisms. Two-component signal transduction systems, composed of sensor protein and cognate response regulator, are commonly used for environmental sensing in bacteria [1]. Environmental stimuli trigger the histidine-aspartate phosphorelay of a phosphate group from an autophosphorylated sensor to a response regulator, resulting in the expression of the particular set of genes necessary for adaptive responses. The PhoRB system of *Escherichia coli* is a well characterized two-component system that senses inorganic phosphate (Pi) concentration in the external milieu [2]. PhoR is a sensor histidine kinase that phosphorylates PhoB, the response regulator, in conditions of low environmental Pi (less than 4 µM in the case of *E. coli*). Phospho-PhoB in turn regulates transcription of the Pho regulon, a large set of genes that are generally involved in phosphate homeostasis. PhoB has been associated with bacterial survival, stress response and virulence [3]. Induction of the Pho regulon has been reported during infections of *Yersinia pestis*, *Erwinia chrysanthemi*, *Listeria monocytogenes* and *Mycobacterium tuberculosis* in diverse models [4]–[8].

The mammalian gut harbors a wide variety of microbes that must sense and respond to changes in nutritional availability, host immunity and microbial composition [9], [10]. Post-operative injury leads to the release of host physiological stress products into the intestinal tract, where they directly activate the molecular circuitry of colonizing nosocomial pathogens, shifting their phenotypes to those of proinflammatory and lethal strains 11,12. It has also been shown that intestinal Pi levels decrease after surgical operations such as 30% hepatectomy [13]. Operative injury-induced intestinal Pi depletion shifted the phenotype of *Pseudomonas aeruginosa* to express enhanced virulence in mice, resulting in exotoxin A permeability and death [11]. Pi repletion protected mice from gut-derived sepsis by *P. aeruginosa*. *Bacteroides*, a gram-negative obligate anaerobe, is one of the most predominant genera of gut microbiota. *B. fragilis* is among the more virulent species in the genus *Bacteroides*, and is frequently isolated from intra-abdominal infections such as peritonitis and peritoneal abscess [14]–[16]. *Bacteroides* infections often occur after intra-abdominal surgery. It is possible that alterations of intestinal parameters like Pi concentration and other environmental cues at extra-intestinal sites induce a shift from gut symbiosis to pathogenicity.

In this study, we identified the two-component signal transduction system corresponding to PhoRB in the sequenced *B. fragilis* strain YCH46 and evaluated the role of the *B. fragilis* PhoB on virulence in the intra-abdominal cavity and survival in peritoneal abscesses. The data indicated that PhoB shapes part of the molecular circuitry used by *B. fragilis* for survival in stressful environments.

## Materials and Methods

### Bacterial Strains and Growth Conditions

The bacterial strains and plasmids used in this study are listed in Table 1. *B. fragilis* strains were grown anaerobically at 37°C in Gifu Anaerobic Medium (GAM; Nissui Pharmaceutical Co., Tokyo, Japan) or on GAM agar plates using the AnaeroPack System (Mitsubishi Gas Chemical Co., Inc., Tokyo, Japan) or an anaerobic chamber conditioned with mixed gases (N_2_, 80%; CO_2_, 10%; and H_2_, 10%). To simulate the Pi-limiting conditions, defined minimal media (DMM) [17] was supplemented with varying concentrations of KH_2_PO_4_ (final concentration: 6.6, 0.066, and 0.0066 mM). *E. coli* strains were grown aerobically at 37°C in Luria-Bertani (LB) broth or on LB agar plates. If necessary, antibiotics were added to the media at the following concentrations: ampicillin, 50 µg/ml; cefoxitin, 50 µg/ml; erythromycin (Em), 10 µg/ml; and tetracycline, 10 µg/ml.

**Table 1:** Bacterial strains and plasmids.

Strain or plasmid	Relevant genotype or description	Reference or source
**Strains**		
* Escherichia coli*		
DH5α	F^−^ Φ80d*lacZ*ΔM15 Δ(*lacZYA-argF*)U169 *deoR recA*1 *endA*1 *hsdR*17(r_K_^-^ m_K_^+^) *phoA supE*44 λ^-^ *thi*-1 *gyrA*96 *relA*1	Laboratory strain
BL21	F^−^ *ompT hsdS_B_* (r_B_^−^m_B_^−^) *gal dcm*	Laboratory strain
* Bacteroides fragilis*		
YCH46	Clinical isolate, parental strain for all deletion mutants	37
TCSM1575 (ΔBF1575)	YCH46 mutant with deletion of BF1575	This study
TCSM1576 (ΔBF1576)	YCH46 mutant with deletion of BF1576	This study
TCSM2185 (ΔBF2185)	YCH46 mutant with deletion of BF2185	This study
**Plasmids**		
pKK100	Suicide vector for *Bacteroides*; Tc^R^ in *E. coli*, Em^R^ in *Bacteroides*	19
pLYL05	*E. coli-Bacteroides* shutle vector; Amp^R^ in *E. coli*, Cfx^R^ in *Bacteroides*	38
pLYL05-Exp	*Bacteroides* expression vector, IS*1224*/*cepA* hybrid promoter (39), restriction sites (*Nde* I, *Not* I, *Xba* Iand *Sal* I), and the transcription terminator of BF1719 cloned into the *Kpn* I/*Sph* I site of pLYL05	This study
pLYL1576	BF1576 amplified with primers phoB-NdeI and phoB-XbaI from *B. fragilis* strain YCH46 clonedinto the *Nde* I/*Xba* I site of pLYL05-Exp	This study
pGEX-6P-1	GST gene fusion vector, Amp^R^	GE Healthcare
pGEX-phoB	BF1576 amplified with primers phoB-BamHI and phoB-R from *B. fragilis* strain YCH46 cloned into the*Bam*H I/*Sma* I site of pGEX-6P-1	This study

### *B. fragilis* Gene Deletion

Deletion mutants for the *phoR* homolog (BF1575), *phoB* homolog (BF1576), or BF2185 were constructed in the *B. fragilis* strain YCH46 by removing the internal segment of each target gene. Briefly, DNA fragments upstream and downstream of the region being deleted were separately PCR-amplified and fused by a second PCR amplification via an overlapping regions incorporated into the primer sequences. The resultant PCR products were ligated into pKK100 [18], [19]. The targeting plasmids were electroporated into *B. fragilis* strain YCH46 as described previously [18], [19]. The diploids, in which targeting plasmid integrated into the chromosome via a single genetic crossover, were selected on GAM agar plates containing Em. The diploids were grown in GAM broth, spread on nonselective GAM agar plates, and replica plated to GAM agar plates containing Em to screen for mutants that resolved the diploid through the second homologous recombination. Em-sensitive colonies were selected, and the presence of the appropriate deletion was checked by PCR with primer pairs that flanked the deletion site (Table S1). The *phoB* gene was amplified using the *B. fragilis* YCH46 genome as the template and cloned into the modified *E. coli*-*Bacteroides* shuttle plasmid pLYL05-Exp (Table 1), generating pLYL1576. pLYL05-Exp or pLYL1576 were introduced into the *phoB* deletion mutant for complementation analyses. Synthetic oligonucleotide primers were purchased from Sigma-Aldrich Japan Co., Ltd. (Tokyo, Japan). The nucleotide sequences of all of the oligonucleotide primers used in this study are listed in Table S1. DNA sequencing was performed on an ABI Prism 3100 Genetic Analyzer (Applied Biosystems) using the ABI Prism BigDye Terminator Cycle Sequencing Ready Reaction Kit (version 1.1; Applied Biosystems).

### Growth Monitoring in Pi-limiting Conditions

*B. fragilis* strains were grown anaerobically in 10 ml of GAM broth at 37°C overnight (stationary phase). The cells were collected by centrifugation at 4°C, washed three times with 10 ml of DMM supplemented with 0.0066 mM KH_2_PO_4,_ and resuspended in 10 ml of the same medium. The cell suspension (100 µl) was then inoculated into 10 ml of pre-warmed DMM (37°C) supplemented with varying concentrations of KH_2_PO_4_ (6.6, 0.066, or 0.0066 mM) and incubated anaerobically at 37°C. Growth was monitored over time by measuring the optical density at 600 nm. All manipulations were carried out in an anaerobic chamber. The doubling time of each strain was calculated from the optical densities at the two points of exponentially growing phase.

### RNA Isolation and Quantitative PCR (qPCR)

*B. fragilis* cells grown to stationary phase in GAM were washed three times with DMM containing 0.0066 mM of KH_2_PO_4_ and diluted 100-fold with DMM plus 6.6 mM or 0.0066 mM of KH_2_PO_4_. These dilutions were grown at 37°C under anaerobic conditions. Total RNA was extracted from mid-logarithmic phase cultures (OD_660_; 0.4–0.6) using the hot-phenol method [20]. The RNA was further purified using an RNeasy CleanUp Kit (Qiagen) and treated with TURBO DNA-*free* (Ambion) to remove contaminating DNA. Total RNA (500 ng) was reverse transcribed using a SYBR ExScript RT-PCR Kit (Takara Shuzo Co., Ltd., Otsu, Japan) with random hexamers at 42°C for 15 min. Reverse transcription was terminated by heating the mixtures at 95°C for 2 min. The cDNA products were subsequently amplified using SYBR Premix Ex Taq (Takara) under the following conditions: preheating at 95°C for 10 s and 40 cycles of 95°C for 5 s and 60°C for 34 s in an ABI PRISM 7500 (Applied Biosystems). All samples were run in triplicate. Threshold cycle values were normalized with the levels of *rpoD* transcripts, and the changes were calculated by the 2^−ΔΔ*CT*^ method [21].

### Microarray Analysis

We employed a *B. fragilis* YCH46 DNA microarray from NimbleGen Systems, which includes 4,527 target genes with at least 8 unique probes consisting of 60-mer synthetic oligonucleotides for each gene. The cDNA synthesis, hybridization, and scanning were performed by NimbleGen. Microarray data were analyzed by quantile normalization and robust multiarray averaging [22]. The normalized data were processed with ArrayStar software (DNASTAR). Samples were filtered to identify differential expression (over 4-fold change during Pi starvation compared to Pi excess). The Student’s *t* test for the analysis of the mean log ratios of two samples and the subsequent Bonferroni adjustment for multiple testing were applied as rigorous criteria for significant changes in signal intensity. Changes with a *p*-value less than 0.05 (*p*<0.05) were considered statistically significant.

### Expression and Purification of Recombinant PhoB

BF1576 was amplified by PCR and cloned into the pGEX-6P-1 expression vector (GE Healthcare) via fusion to glutathione-S-transferase (GST) gene, generating the plasmid pGEX-phoB (Table 1). *E. coli* strain BL21 harboring pGEX-phoB was grown in 100 ml of LB broth containing sorbitol (0.5 M) and ampicillin (50 µg/ml) at 37°C. IPTG was added to the culture at a final concentration of 0.1 mM when OD_660_ reached 0.3. The cells were further incubated at 25°C overnight. The cells were collected by centrifugation and resuspended with Cell Lytic B (Sigma) containing a protein inhibitor cocktail, lysozyme (200 µg/ml) and DTT (1 mM). Following a 15-min incubation at room temperature, the lysate was sonicated on ice and centrifuged at 13,000×*g* for 10 min at 4°C. The supernatant was incubated with glutathione sepharose 4B slurry (GE Healthcare) overnight at 4°C. The resin was washed five times with PBS-T wash buffer (1× phosphate-buffered saline, 0.5% Triton X-100). Proteins bound to the resin were eluted with 200 µl of glutathione elution buffer (50 mM Tris-HCl [pH8.0], 10 mM glutathione). Glutathione elution buffer was replaced by EMSA buffer (50 mM Tris-HCl [pH7.5], 50 mM KCl, 10 mM MgCl_2_, 0.5 mM EDTA, 10% glycerol) using Zeba Desalt Spin Columns (Thermo Scientific). The purity of recombinant PhoB was checked by SDS-PAGE.

### Electrophoretic Mobility Shift Assay

Recombinant PhoB and 10 ng of bait DNA were mixed in binding buffer (50 mM Tris-HCl [pH7.5], 50 mM KCl, 10 mM MgCl_2_, 0.5 mM EDTA) and incubated for 30 min at 25°C. The predicted promoter regions of *pstC* (BF2756) and capsular polysaccharide biosynthesis loci B (PS B, BF1828-BF1848) and E (PS E, BF2566-BF2586), or internal fragment of BF3397 were amplified by PCR. The amplicons were purified using a QIAquick Gel Extraction Kit (Qiagen) after agarose gel separation and used as bait DNA. After incubation, the samples were subjected to electrophoresis on a 5% polyacrylamide gel and visualized using the SYBR Green I Nucleic Acid Gel Stain Kit (Invitrogen).

### Peritoneal Abscess Model

Five male C57BL/6J Jcl mice (7 weeks old) were injected intra-peritoneally with 0.2 ml of an inoculum (2.0×10^8^ colony-forming unit each) that was prepared by mixing *B. fragilis* cell suspensions (wild type, ΔBF1576, ΔBF1576 complemented with pLYL1576, or a combination of wild type and ΔBF1576) with the supernatant of an autoclaved rat fecal suspension at a 1∶1 (vol/vol) ratio. The autoclaved rat fecal suspension did not induce abscesses when injected alone into the mouse peritoneal cavity, and was used as an adjuvant for abscess formation. Mice were sacrificed at 3, 7, or 14 days after challenge, and abscess formation was evaluated. The incidence of peritoneal abscess, the number of abscesses and surviving *B. fragilis* were compared. Prior to dissection of the peritoneum, 10 ml of PBS (pH7.4) was injected into the peritoneal cavity and then recovered. The number of infiltrated inflammatory cells in the recovered fluid was counted using a hemocytometer. The surviving cell number inside the peritoneal abscesse was determined as follows. The collected abscesses were weighed and homogenized in 10 volumes of autoclaved PBS (pH7.4) using Teflon glinder. Serial dilutions were made with PBS (pH7.4) and the appropriate dilutions were spread onto GAM agar plate. The plates were incubated anaerobically at 37°C for 48 h and the numbers of colony grown on the plates were counted. Wild type *B. fragilis* and the ΔBF1576 mutant were discriminated using colony PCR with a primer pair encompassing the deletion site in ΔBF1576 (Table S1), when necessary. At least, 96 colonies per abscess were screened.

### Competitive Intestinal Colonization Model

Three male germ-free BALB/c mice (8 weeks old) were orally inoculated by gavage with the mixture of wild type and ΔBF1576 *B. fragilis* strain (2.0×10^8^ colony-forming unit each). Feces were collected periodically (7 and 14 days after challenge), and appropriate dilutions of the fecal suspensions were spread on GAM agar plates. Colony PCR was performed with the primer pair encompassing the deletion site in ΔBF1576 to compare their population levels of wild and the mutant *B. fragilis* strains in the mouse intestines. In this experiment, mice were kept in a vinyl isolator to maintain the gnotobiotic status.

### Statistical Analysis

The growth rates, qPCR data and the number of abscess were statistically analyzed with Student’s *t* test. The incidence of peritoneal abscess and wild-type/*phoB* mutant ratios in the intestines and abscesses were analyzed with Fisher’s exact test and Chi-square test, respectively. Changes were considered to be significantly different when the *p*-values were less than 0.05.

### Microarray Accession Numbers

Microarray data have been deposited in the Gene Expression Omnibus database (www.ncbi.hlm.nih.gov/projects/geo) under the following accession numbers: normalized data, GSE27439; platform, GPL13213; and raw data files, GSM678272 to GSM678275.

### Ethics

All animal experiments were performed according to the guidelines for animal experiments at the University of Tokushima. The experimental design was approved by the Animal Experiment Committee of the University of Tokushima.

## Results

### Identification of a PhoRB System in *B. fragilis*

The genome of sequenced *B. fragilis* strain YCH46 contains 70 two-component signal transduction systems (TCS), which include orphan kinases and response regulators (Table S2). A BLAST search was performed to identify a TCS of *B. fragilis* that corresponds to PhoRB in other bacteria. Amino acid sequences of *E. coli* PhoR or PhoB were used as queries against the *B. fragilis* genome. The proteins encoded by BF1575 and BF1576 showed the best similarities to *E. coli* PhoR and PhoB, respectively (39% and 29% identity over 50% alignment length, respectively). TCS proteins encoded by BF2185 and BF2186 showed the second highest homologies to PhoRB of *E. coli*. PhoB regulates the expression of a phosphate-specific transport (Pst) system to acquire Pi [3]. The Pst system is composed of five components encoded within the *pstSCAB*-*phoU* operon. According to the gene annotation, in the *B. fragilis pst* operon, *pstS* is located just upstream of the *pstCAB*-*phoU* operon (BF2756-BF2753) in a head-to-head manner.

**Figure 1:**
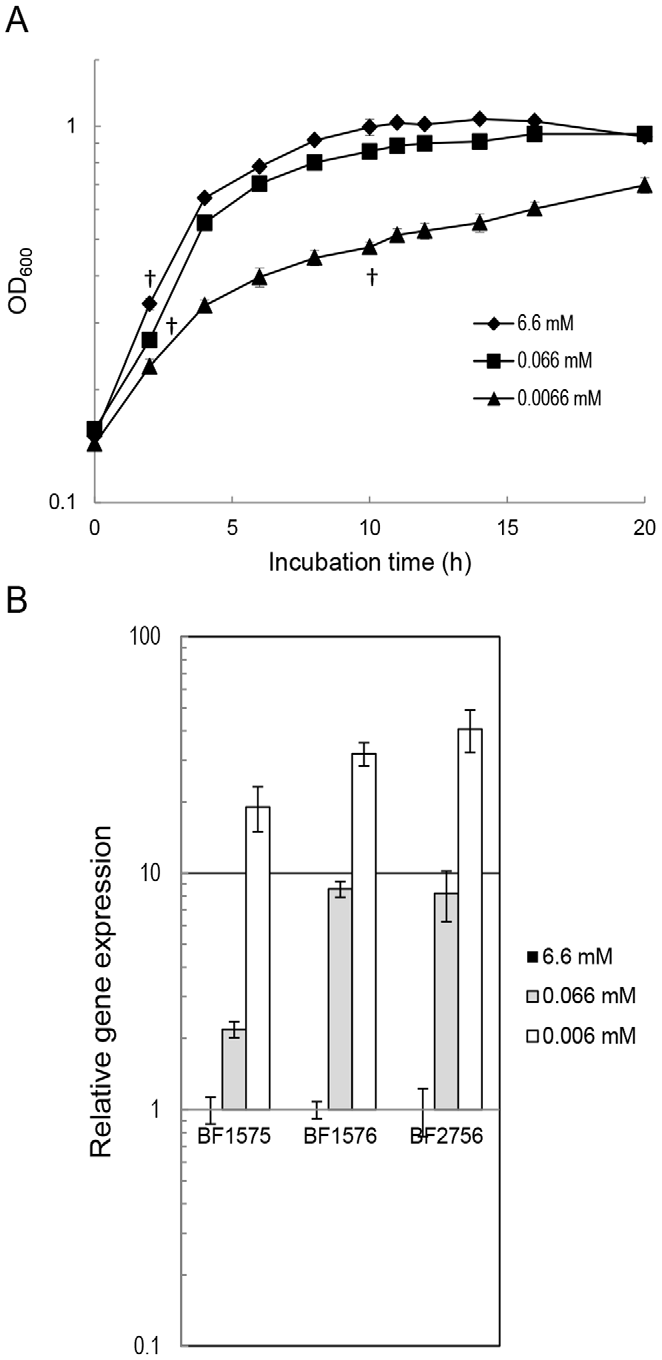
Response of *B. fragilis* to Pi limitation. (A) Growth curve in DMM supplemented with varying concentrations of KH_2_PO_4_. *B. fragilis* strain YCH46 was grown anaerobically in DMM supplemented with 6.6 mM, 0.066 mM, or 0.0066 mM of KH_2_PO_4_ at 37°C for 20 h. Optical densities of the cultures at 600 nm were measured over time. Data presented are the mean ± standard deviations of three independent cultures. (B) qPCR analysis of BF1575, BF1576, and BF2756. Total RNA was collected at mid-logarithmic phase (indicated by † in panel A). Expression levels of BF1575 (*phoR* homolog), BF1576 (*phoB* homolog), and BF2756 (*pstC* homolog) in wild type *B. fragilis* strain YCH46 were measured and normalized with transcriptional levels of *rpoD*. The transcriptional level of each gene under Pi limitation (0.066 mM or 0.0066 mM) is shown relative to that in DMM supplemented with 6.6 mM KH_2_PO_4_ (Pi-rich media).

*B. fragilis* strain YCH46 was grown in DMM supplemented with varying concentrations of KH_2_PO_4_ (final concentration of 6.6, 0.066, or 0.0066 mM). As shown in Figure 1A, a decrease in Pi from 6.6 mM to 0.066 mM did not result in the growth retardation (the doubling times; 2.14±0.08 h vs 1.93±0.03 h). Clear growth retardation was observed when the Pi concentration was limited to 0.0066 mM (the doubling time; 3.85±0.50 h). Based on this result, growth media supplemented with 0.0066 mM KH_2_PO_4_ was used as the Pi-limiting condition in subsequent experiments. RNA was extracted from mid-logarithmic phase cultures and qPCR was performed for BF1575, BF1576, and *pstC* (BF2756). As shown in Figure 1B, the expression levels of these genes were negatively correlated with Pi level and were elevated over 10 fold compared with those assayed in Pi-rich media (6.6 mM of KH_2_PO_4_).

**Figure 2:**
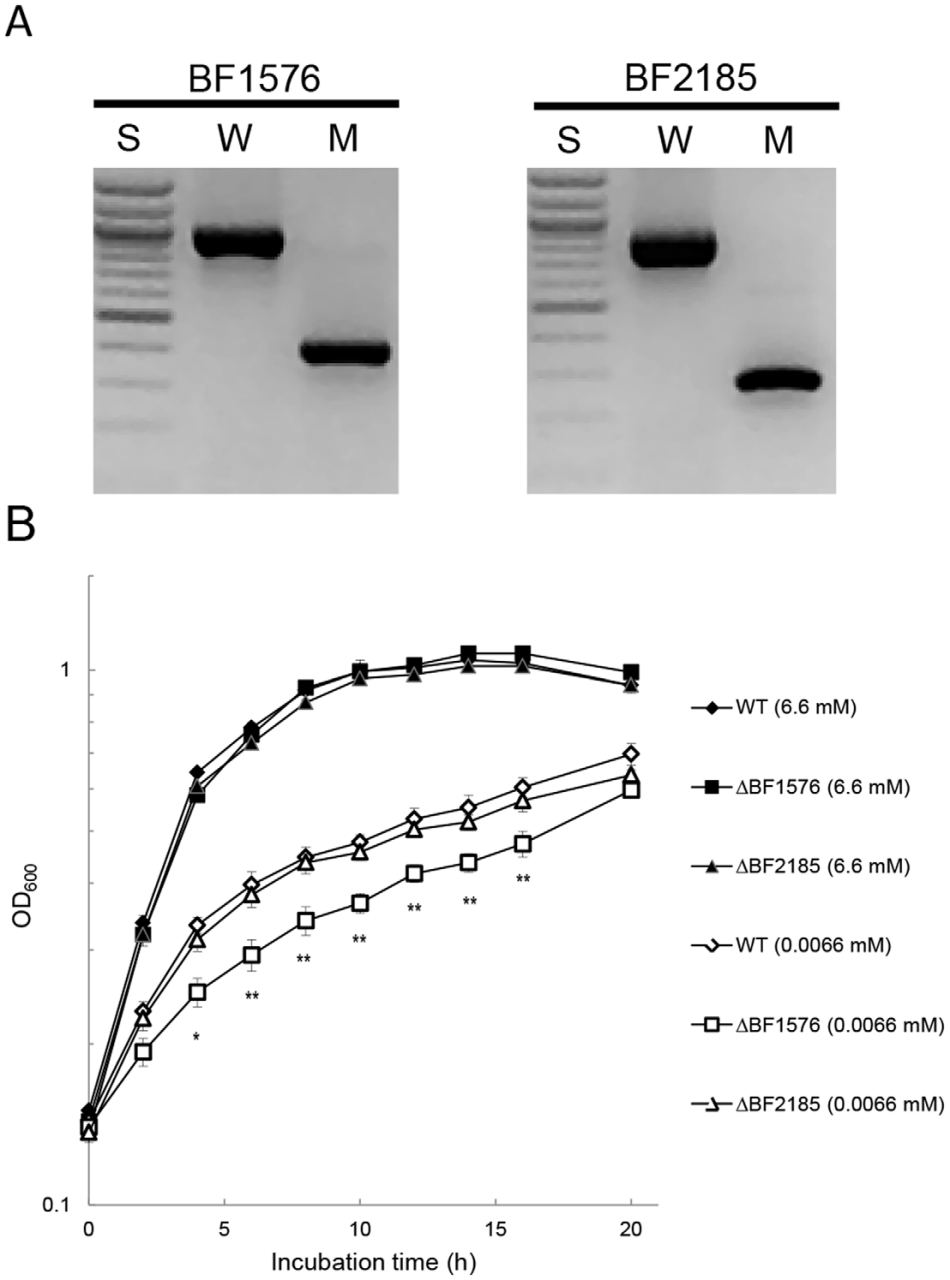
Effect of BF1576 deletion on the growth in Pi-limiting media. (A) Construction of ΔBF1576 and ΔBF2185 strains. Internal deletion of each gene was confirmed by PCR using a primer pair encompassing each deletion site. W, wild type *B. fragilis*; M, mutant form of indicated gene; S, 100-bp ladder size markers. (B) Growth of wild type (WT), ΔBF1576 and ΔBF2185 strains under Pi-rich and Pi-limiting conditions. WT (diamonds), ΔBF1576 (squares), and ΔBF2185 (triangles) strains of *B. fragilis* were grown anaerobically in DMM supplemented with 6.6 mM (closed symbols) or 0.0066 mM (open symbols) of KH_2_PO_4_. Growth was measured at OD_600_. Data presented are the means ± standard deviations of triplicate cultures. **Significantly different from wild type (*p*<0.01), *Significantly different from wild type (*p*<0.05).

**Figure 3:**
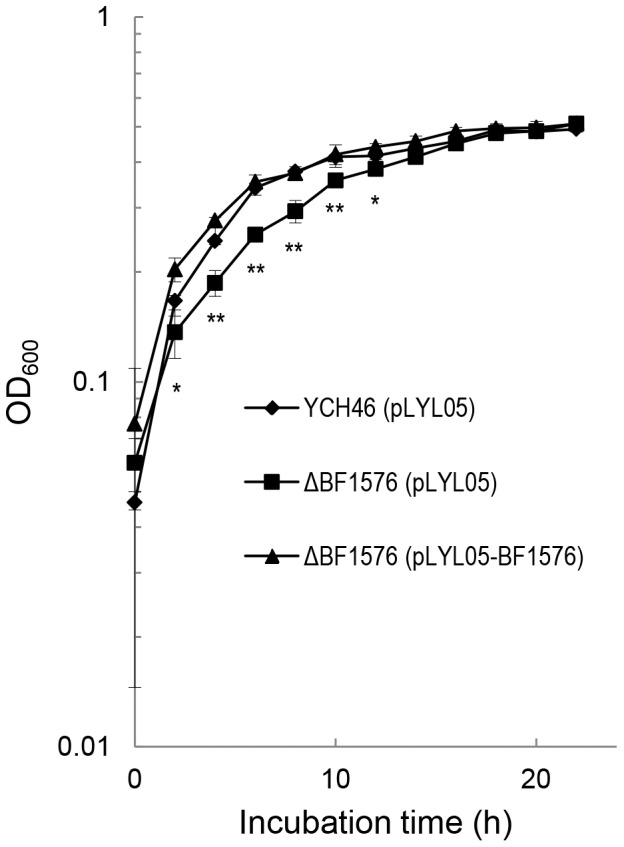
Repletion of BF1576 by plasmid complementation. *B. fragilis* wild type strains harboring pLYL05-Exp and the ΔBF1576 strain harboring pLYL05-Exp or pLYL1576 were grown anaerobically in a limiting amount of Pi (0.0066 mM of KH_2_PO_4_) in DMM supplemented with 50 µg/ml of cefoxitin. Growth was measured at OD_600_. Data were calculated from triplicate cultures and expressed as means ± standard deviations. **Significantly different from the ΔBF1576 strain harboring pLYL05-Exp (*p*<0.01), *Significantly different from the ΔBF1576 strain harboring pLYL05-Exp (*p*<0.05).

**Figure 4:**
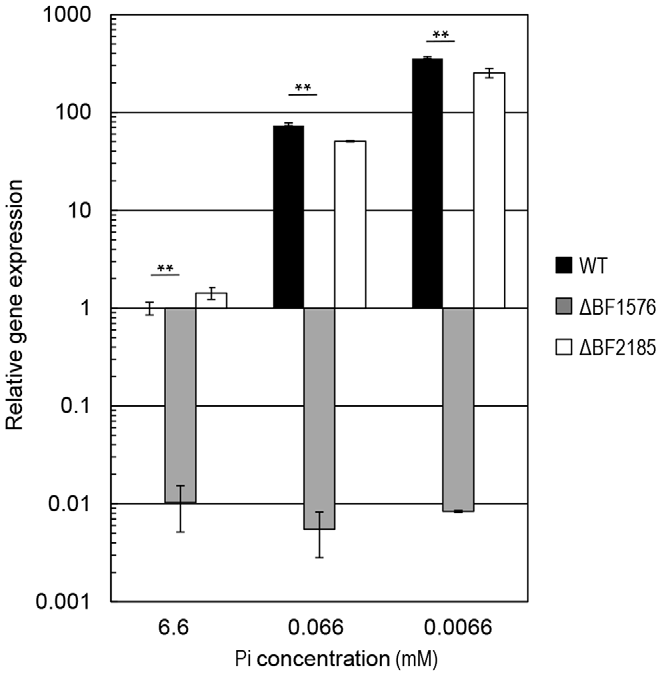
Induction of *pstC* expression in response to Pi limitation. Wild type, ΔBF1576, and ΔBF2185 strains of *B. fragilis* were cultured in DMM supplemented with varying concentrations of Pi as indicated. Total RNA was extracted from mid-logarithmic phase cultures, and *pstC* expression levels were compared by qPCR. The transcriptional level of *pstC* was normalized with that of *rpoD* and shown relative to that of a wild type strain in DMM supplemented with 6.6 mM KH_2_PO_4_ (Pi-rich media). Black column: wild type; gray column: ΔBF1576; white column: ΔBF2185. **Significantly different from the wild type strain (*p*<0.01).

BF1576 was disrupted (ΔBF1576) to determine the role of the *phoB* homolog in adaptation to Pi limitation (Figure 2A). BF2185 encodes a protein with the second highest homology to *E. coli* PhoB. A BF2185 deletion mutant (ΔBF2185) was constructed for comparison to ΔBF1576. Growth of the ΔBF1576 strain was similar in Pi-rich media (the doubling time; 2.31±0.02 h) but slower than that of the wild type and ΔBF2185 strains when they were cultured in Pi-limited media (0.0066 mM of KH_2_PO_4_) (Figure 2B); the doubling times of ΔBF1576, wild type and ΔBF2185 were 5.88±1.21 h, 3.85±0.50 h and 4.12±0.46 h, respectively. Complementation of the ΔBF1576 strain with a plasmid harboring BF1576 restored the growth of this mutant (the doubling time; 4.26±0.40 h) to wild type level in DMM supplemented with 0.0066 mM of KH_2_PO_4_ (Figure 3). Under Pi limitation, PhoB binds the “Pho box” regulatory element in the promoter of the *pst* operon and activates expression of the *pst* genes. A comparison of the expression of *pstC* in the wild type and mutant strains in low Pi revealed that the ΔBF1576 strain showed a trace amount of *pstC* expression while the expression level in the ΔBF2185 strain was equivalent to that of the wild type strain (Figure 4). These results indicated that BF1576 encodes PhoB in *B. fragilis*.

**Figure 5:**
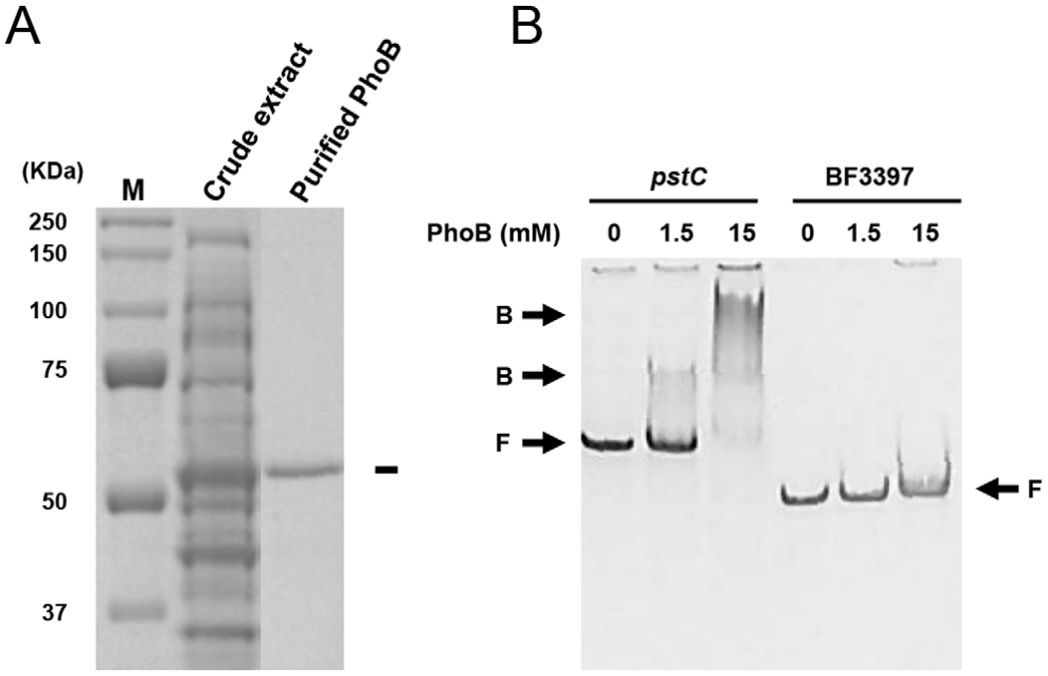
PhoB binds to *pstC* promoter. (A) Expression and purification of recombinant *B. fragilis* PhoB in *E. coli*. M, molecular size markers. (B) Binding of PhoB to *pstC* promoter. Recombinant PhoB (1.5 or 15 µM of final concentration) was mixed with the amplified promoter region of *pstC* or internal region of BF3397, and their interaction was evaluated by electrophoretic motility shift assay. Zero denotes that no protein was added to the reaction mixture. B: PhoB-bound probes; F: free probes.

To confirm the direct association of PhoB with *pstC* expression, the protein was overexpressed in *E. coli* and purified (Figure 5A). The binding of the recombinant PhoB to the *pstC* promoter was assessed by electrophoretic motility shift assay (EMSA). EMSA demonstrated the direct binding of PhoB to the promoter of *pst* operon (512-bp fragment) but not to another DNA fragment used as a control (290-bp internal fragment of BF3397) (Figure 5B).

### Pho Regulon in *B. fragilis*

**Figure 6:**
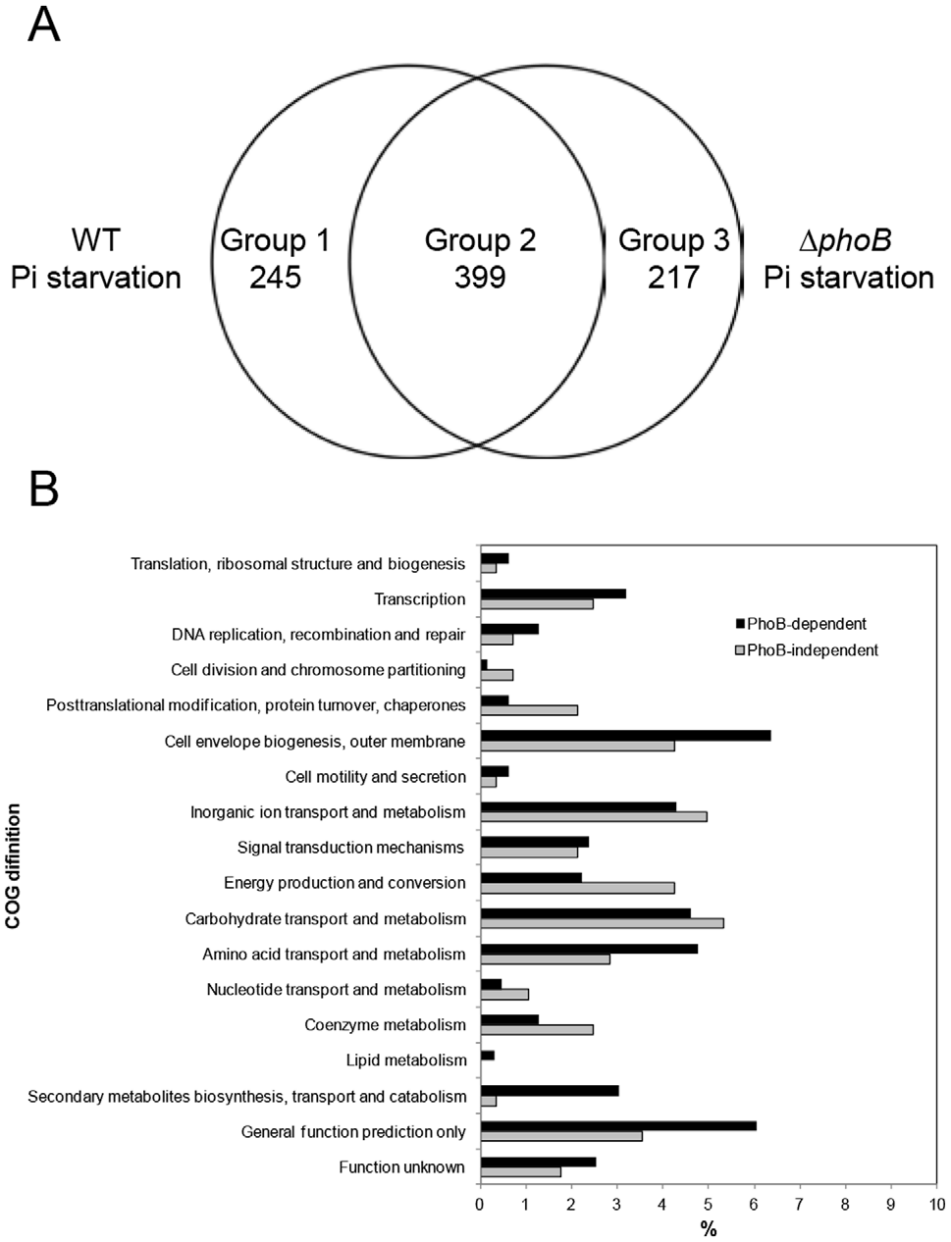
PhoB-dependent genes in *B. fragilis*. (A) Classification of genes whose expression levels were altered more than 4-fold under Pi limitation (0.0066 mM of KH_2_PO_4_) in wild type and Δ*phoB* mutant strains, compared to levels in wild type in the presence of sufficient Pi (6.6 mM of KH_2_PO_4_). Left and right circles indicate the differentially expressed genes of wild type and Δ*phoB* mutant strains with expressions that were altered over 4-fold under Pi limitation. Group 1: includes 245 PhoB-dependent Pi-response genes. Group 3∶217 PhoB-dependent genes unrelated to Pi response. Group 2 contains two subgroups. Group 2a: 276 PhoB-independent Pi-response genes (fold change in expression in response to Pi-limitation was similar between wild type and Δ*phoB* mutant strains); Group 2b: 123 PhoB-dependent Pi response genes (the extent of change in expression in response to Pi-limitation was different >2-fold between wild type and Δ*phoB* mutant strains). (B) COG classification of PhoB-dependent (black bar) and PhoB-independent (white bar) genes that are differentially expressed during Pi starvation. The percentage of genes classified into each COG category is relative to the total number of genes in each group. Fifty-five percent of the PhoB-dependent (585 genes) and 60% of the PhoB-independent (276 genes) groups could not be assigned to a COG category (that data are not included in this figure).

Transcriptome analysis was performed to identify the regulatory circuit of PhoB in *B. fragilis*. Compared to the wild type expression profile in Pi-rich media (6.6 mM of KH_2_PO_4_), the expression of 644 (wild type) and 616 (Δ*phoB* mutant) genes changed more than 4-fold under Pi-limiting conditions (Figure 6A). Of these, the expression levels of 399 genes were altered in both wild-type and the Δ*phoB* mutant (group 2 in Figure 6A), although some of the genes underwent different degree of transcriptional changes depending on the strain (e.g., BF2091 and BF2391 in Table 2). Group 1 in Figure 6A included 245 genes with expression levels that changed more than 4-fold under Pi-limitation (6.6 mM to 0.0066 mM of KH_2_PO_4_) in wild type, but did not change in the Δ*phoB* mutant, which corresponded to PhoB-dependent Pi response genes. In contrast, Group 3 included 217 genes whose expression levels in the Δ*phoB* mutant were affected more than 4-fold under Pi limitation but were unchanged in the wild type strain, indicating that they were PhoB-dependent genes that were unrelated to the Pi response. The genes classified as Group 2 were divided into two subgroups. Group 2a contained 276 PhoB-independent Pi-response genes (the magnitude of changed expression levels in response to Pi-limitation was similar between the wild type and Δ*phoB* mutant strains) and Group 2b had 123 PhoB-dependent Pi response genes (the extent of change in expression in response to Pi-limitation was different >2-fold between wild type and Δ*phoB* mutant strains). The 585 genes classified into Groups 1, 2b, and 3 were defined as Pho-regulon genes. Representative genes with significantly altered expression patterns under Pi limitation are listed in Table 2. Of these, PhoB-dependent genes included those associated with Pi metabolism such as PstSCAB, alkaline phosphatase (BF0480 and BF1756), polyphosphate kinase (BF2645), exopolyphosphatase (BF2646), a putative phosphate/sulphate permease (BF3715) and acid phosphatase (BF4541). In addition to Pi metabolism, genes involved in capsular polysaccharide biosynthesis (PS A, B and E), ferrous iron transport (BF1417), protein repair and chaperon activity, and DNA repair and recombination were also regulated in a PhoB-dependent manner. These data suggest that PhoB may be involved in *B. fragilis* virulence, the stress response and iron homeostasis, as well as in Pi metabolism. To assess the effect of KH_2_PO_4_ limitation on pH of the culture media, we checked the pH of 12-h and 24-h culture of wild or *phoB* mutant strain. The pH of these cultures were kept around 7.6, indicating the effect of acid stress could be ruled out (data not shown). The differentially expressed genes during Pi starvation were assigned to the Cluster of orthologous gene (COG) classification (Figure 6B). The PhoB-regulated function preferentially distributed into Transcription; DNA replication, recombination and repair; Cell envelope biogenesis, outer membrane; Amino acid transport and metabolism; and Secondary metabolites biogenesis, transport and catabolism.

**Table 2:** Representative list of differentially expressed genes in wild type and Δ*phoB B. fragilis* strains in Pi-limiting conditions.

Gene	Function	Fold change a)		Group b)
		WT	Δ*phoB*	
***Inorganic ion metabolism and transporter***				
BF0375	polyphosphate-selective porin O	+3.55	−2.70	2b
BF0480	alkaline phosphatase III precursor	+6.37	−	1
BF0578	potassium-transporting ATPase subunit A	+121	+161	2a
BF0579	K+-transporting ATPase B chain	+151	+238	2a
BF0580	K+-transporting ATPase C chain	+163	+261	2a
BF0582	osmosensitive K+ channel histidine kinase	+88.5	+128	2a
BF1417	putative ferrous iron transport protein	−	−8.18	3
BF1756	alkaline phosphatase	+12.2	−	1
BF2091	cation efflux system protein	+22.3	−4.69	2b
BF2092	CzcA family cation efflux system protein	+22.4	−5.67	2b
BF2289	putative polyphosphate-selective porin O	−3.16	+1.37	2b
BF2392	putative cation efflux pump	+80.0	+21.7	2b
BF2393	putative cation efflux pump	+61.7	+17.1	2b
BF2604	multidrug efflux pump BexA	−4.76	−	1
BF2645	polyphosphate kinase	+24.4	−	1
BF2646	putative exopolyphosphatase	+42.1	−	1
BF2753 (*phoU*)	putative transcriptional regulator for phosphate uptake	−	−16.9	3
BF2754 (*pstB*)	putative phosphate transport ATP-binding protein	−	−353	3
BF2755 (*pstA*)	putative ABC transporter permease protein	−	−542	3
BF2756 (*pstC*)	putative ABC transporter permease protein	−	−428	3
BF2757 (*pstS*)	phosphate ABC transporter phosphate-binding protein	+4.58	−103	2b
BF3145	multidrug resistance ABC transporter	+4.32	−	1
BF3309	sugar transporter	+17.7	+15.5	2a
BF3350	putative glucose/galactose transporter	+13.3	+5.43	2b
BF3714	polyphosphate kinase	+5.22	+4.94	2a
BF3715	putative phosphate/sulphate permeases	−	−52.2	3
BF3721	cation efflux system protein	−	−5.76	3
BF3722	AcrB/AcrD/AcrF family cation efflux system protein	−	−5.69	3
BF4130	putative phosphate ABC transporter phosphate-binding component	+4.10	−	1
BF4134	MotA/TolQ/ExbB proton channel	+3.78	−1.42	2b
BF4348	putative calcium-transporting ATPase	−2.42	+1.68	2b
BF4351	putative Na+-dependent phosphate transporter	−101	−	1
BF4541	acid phosphatase	+328	−	1
***Nucleic acid repair, recombination and metabolism***				
BF1683	Holliday junction resolvase-like protein	−	−4.15	3
BF1862	SOS mutagenesis and repair protein UmuC	+7.84	−	1
BF1863	error-prone repair: SOS-response transcriptional repressor UmuD	+11.2	−	1
BF1883	putative AAA family ATPase	+4.42	−	1
BF2939	2′,3′-cyclic nucleotide 2′-phosphodiesterase	−	−4.37	3
BF3503	uracil-DNA glycosylase	+11.0	−	1
BF4422	DNA-binding protein HU-beta	−	−5.00	3
***Protein repair and chaperons***				
BF1205	endopeptidase Clp ATP-binding chain B	+9.42	+6.60	2a
BF1225	molecular chaperone DnaK	+12.5	+7.93	2a
BF1742	chaperone protein DnaJ	+6.75	+4.06	2a
BF1743	GrpE protein	+8.08	+4.23	2a
BF2021	peptidyl-prolyl cis-trans isomerase	−5.05	−	1
BF2409	heat shock protein 90	+16.5	+8.23	2b
BF2625	small heat shock protein	+7.71	−	1
BF3377	putative chaperone DnaJ	+13.3	+5.01	2b
BF3395	chaperonin GroEL	+8.23	+7.10	2a
BF3396	co-chaperonin GroES	+19.3	+12.1	2a
***Cell envelope biogenesis and outer membrane***				
BF0002	putative outer membrane protein TolC	−1.45	+3.39	2b
BF1428 (*upaY*)	transcriptional regulatory protein UpxY homolog in capsular polysaccharide synthesis locus (PSA)	−6.50	−16.4	2b
BF1828 (*upbY*)	transcriptional regulatory protein UpxY homolog in capsular polysaccharide synthesis locus (PSB)	−5.55	+10.0	2b
BF2391	outer membrane efflux protein oprM precursor	+106	+28.4	2b
BF2585 (*upeY*)	transcriptional regulatory protein UpxY homolog in capsular polysaccharide synthesis locus (PSE)	−	−330	3

a) Fold change (relative to wild-type 6.6 mM Pi) was determined from the microarray expression data as described in the Materials and Methods.

b) The definition of each group is described in legend to Figure 6.

**Figure 7:**
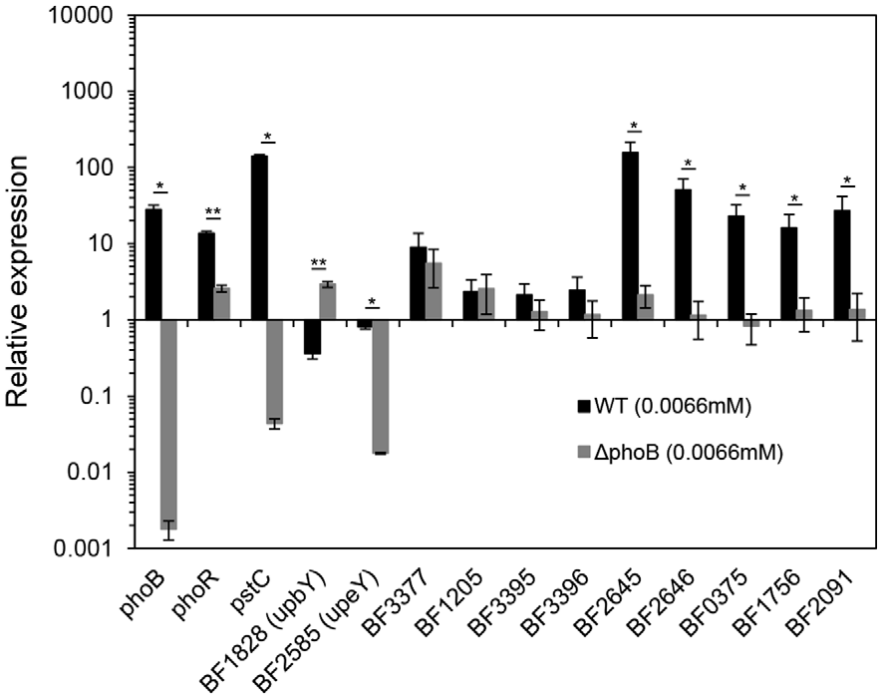
qPCR verification of differentially expressed PhoB-dependent genes identified by microarray analysis. The transcriptional levels of representative genes in wild type (closed column) and *phoB* deletion mutant (open column) under Pi limitation (0.0066 mM of KH_2_PO_4_) is shown in relation to those in wild type under Pi rich condition (6.6 mM of KH_2_PO_4_). Annotation of each gene is as listed in Table 2: BF1576 (PhoB), BF1575 (PhoR), BF2185 (PstC), BF1828 (UpbY, product of the first gene product in PS B), BF2585 (UpbY, product of the first gene product in PS E), BF3377 (DnaJ), BF1205 (endopeptidase Clp), BF3395 (GroEL), BF3396 (GroES), BF2645 (polyphoaphate kinase), BF1576 (alkaline phosphatase), and BF2091 (cation efflux system protein). **Significantly different from the wild type strain, *Significantly different from the wild type strain (*p*<0.05).

To verify the microarray analysis data, qPCR was performed on the representative PhoB regulon genes listed in Table 2, which include stress-response, phosphate metabolism and polysaccharide biosynthesis (PS B and PS E). As shown in Figure 7, the expression profiles correlated well the results from microarray analysis (Table 2).

### Role of PhoB in Peritoneal Abscess Formation

Wild type and Δ*phoB* mutant *B. fragilis* strains were inoculated into the peritoneal cavities of C57BL/6J mice to compare their ability to induce abscess formation. Mice were sacrificed at 3, 7, or 14 days after challenge, and the number of abscesses was counted. One week after challenge, the wild type *B. fragilis* strain formed peritoneal abscesses in 80% of the mice (Table 3). Abscesses were observed in only 40% of the mice tested with the Δ*phoB* mutant. In addition, a total of 13 abscesses were produced by wild type *B. fragilis*, while only 2 abscesses were observed with the Δ*phoB* mutant. Complementation of the Δ*phoB* mutant with a plasmid harboring *phoB* restored the number of abscesses. However, during the early stage of peritoneal infection (3 days after challenge), no difference was found in incidence and abscess number between wild type and Δ*phoB* mutants. The viable cell numbers inside the abscesses were also not significantly different among the strans (Table 4). In addition, inflammatory cell infiltration into peritoneal cavity was similar between the wild type strain (6.20±2.96×10^6^/ml of the recovered fluid) and *phoB* mutant (5.73±2.83×10^6^/ml) at an early stage (3 days) of infection (2.92±1.10×10^6^/ml in autoclaved rat feces alone). These findings indicate that in *B. fragilis*, PhoB plays a role in abscess persistence rather than formation.

**Table 3:** Peritoneal abscess formation by *B. fragilis* strains.

B. fragilis strain	Abscess formation a)					
	Incidence			Number ofabscessb)		
	3 days	7 days	14 days	3 days	7 days	14 days
YCH46(wild type)	5/5	4/5	4/5	3.40±1.34	2.00±1.22	1.40±1.14
ΔBF1576(ΔphoB)	5/5	2/5*	2/5*	3.00±1.58	0.40±0.55*	0.40±0.55
ΔBF1576(pLYL1576)	5/5	4/5	4/5	2.20±0.45	1.60±1.52	0.80±0.45

a) Five C57BL/6J mice were intraperitoneally challenged with *B. fragilis* strains. Mice were sacrificed and abscess formation was evaluated on the indicated days after challenge.

b) Number of abscess in each mouse (mean ± SD) is shown.

* Significant difference from YCH46 (wild type), *p*<0.05.

**Table 4:** Viable *B. fragilis* cell numbers in the peritoneal abscesses *^a^*).

Strains	Viable cell counts (log_10_ cfu/abscess) b)		
	3 days	7 days	14 days
YCH46 (wild type)	6.58±1.12	6.21±1.08	6.24±1.64
*phoB* mutant	6.54±1.14	6.50±0.81	5.91±0.23

a) Each of the strains (ca. 2.0×10^8^ cells) was individually inoculated into the mouse peritoneal cavity to form abscesses. Each of the corrected abscesses was homogenized in PBS, and the serial dilutions were plated on GAM agar plates.

b) Data were mean ± SD. No significant differences were observed.

**Figure 8:**
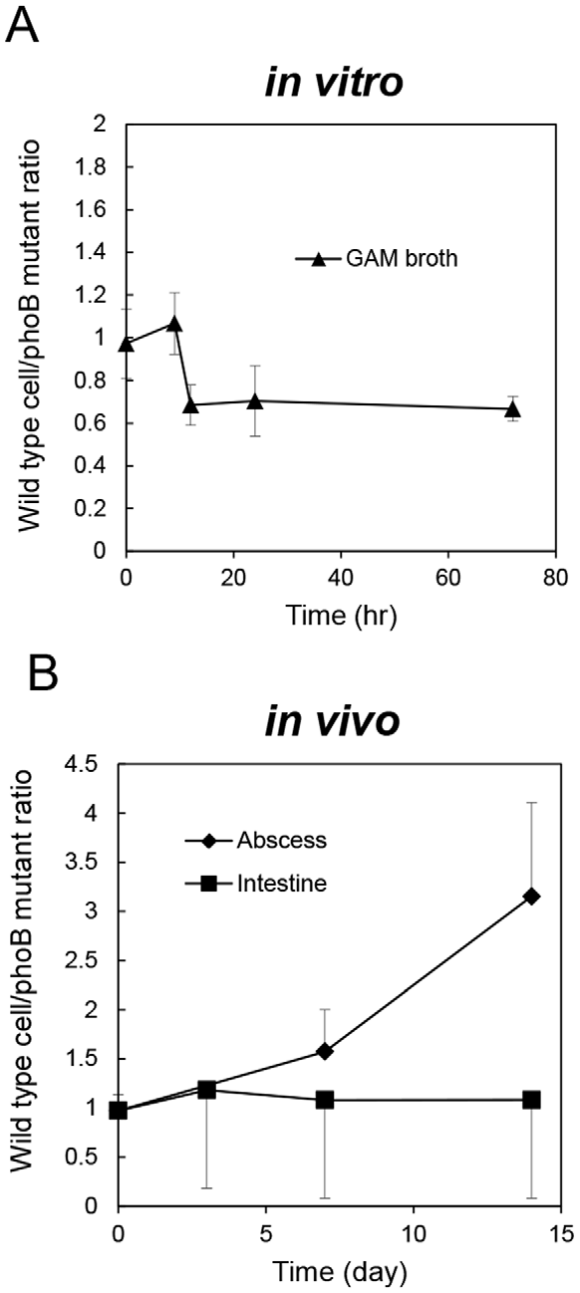
Competitive growth assay of wild type and Δ*phoB B.* * fragilis* strains *in vitro* (A) and *in vivo* (B). These strains were equally mixed and inoculated into GAM broth, mice intestines or the peritoneal cavity. After periodical sampling from liquid culture, feces and abscesses, samples were spread on GAM agar plates, and the population levels of wild type and Δ*phoB B. fragilis* strains were evaluated by discriminating colony PCR. The data were expressed as wild type/Δ*phoB* mutant ratios. The ratios of wild type and *phoB* mutant strains in the intestine and abscess were compared by Chi-square test.

To compare the growth and survival of wild type and Δ*phoB B. fragilis* strains under various conditions, these strains were equally mixed and inoculated into GAM broth, mice intestines or the peritoneal cavity. After periodical sampling from liquid culture, feces and abscesses, samples were spread on GAM agar plates, and the population levels of wild type and Δ*phoB B. fragilis* strains were evaluated by discriminating colony PCR. Deletion of *phoB* did not affect growth in rich media (GAM broth), and the ratios of wild type to *phoB* mutant strains were 0.67 to 1.07 until 72-h cultivation (Figure 8A). The *phoB* deletion also had no effect on intestinal colonization, and equivalent populations of each cell type were maintained until two weeks after inoculation into the gut of eight-week-old male BALB/c germ-free mice (Figure 8B). In contrast, the number of Δ*phoB* mutant cells decreased more rapidly than the number of wild type cells when survival within the peritoneal abscess was compared. The ratio of Δ*phoB* mutant to wild type cells inside the abscess was reduced to one-third at two weeks after intra-peritoneal inoculation (Figure 8B). The ratios of wild type to *phoB* mutant after one and two week inoculation were significantly different (Chi-squre *p* values are 0.0173 and less than 0.0001, respectively). These results indicate that PhoB is involved in the survival of *B. fragilis* in stressful extraintestinal environments.

## Discussion

Microbes inhabit all of the environmentally exposed anatomical sites of the human body. Individual microbial species have adapted uniquely to their preferential niches. Signals present in these microenvironments play key roles in shaping host-microbe interactions. Through these signals, commensal bacteria provide beneficial actions such as digestion of otherwise undigestible nutrients, synthesis of essential vitamins and competitive colonization resistance to foreign pathogenic bacteria (symbiosis). Disruption of these homeostatic signals leads to pathogenic conditions such as overgrowth or submucosal invasion of commensal bacteria, which elicit the host inflammatory response (dysbiosis). The persistence of dysbiosis results in diseases such as inflammatory bowel syndrome [23], [24], cancer [25] and obesity [26]–[29].

The intestinal tract harbors the most densely populated microbiota in the human body. *Bacteroides* is one of the predominant groups of gut commensals [10]. Although *B. fragilis* is one of the most pathogenic species of the genus *Bacteroides* and often causes peritonitis, intra-abdominal abscesses and septicemia [14]–[16], the bacterium is considered to be a keystone microbe that controls the microbial ecosystem in the gut [30]. The two-component signal transduction system (TCS) is a major bacterial environmental sensing system. The whole genome sequence of *B. fragilis* revealed that this anaerobe possesses 70 TCS [31]. They undoubtedly play essential roles in the processing of environmental signals within the gut. However, the environmental signals that induce the TCS are largely unknown. Pi limitation is a well characterized environmental signal sensed by PhoRB, a TCS widely distributed among prokaryotes. Pi depletion after abdominal surgical operations is a risk factor for sepsis caused by gut-derived opportunistic pathogens [11], [12]. In this study, we focused on this stimulus (Pi concentration) to assess the importance of environmental sensing systems on shaping the symbiosis and dysbiosis within the gut.

We identified the PhoRB system in the sequenced *B. fragilis* strain YCH46 and demonstrated that PhoB, as in other bacteria, plays a role in adaptation to Pi-limiting conditions. However, it is unlikely that PhoRB is the sole Pi acquisition system in *B. fragilis* because *phoB* deletion did not result in a complete growth defect (Figure 1A). No difference was observed in the maximum optical density of the culture in Pi-limiting media. In addition to the *pst* operon, PhoB regulates the expression of 585 genes in *B. fragilis*. Of these, 217 are regulated in a Pi-dependent manner (Group 1 in Figure 6A). The remaining 368 genes are regulated independent of Pi concentration (Group 2b and Group 3 in Figure 6A). This group includes several stress response genes and capsular polysaccharide biosynthetic genes (PS A, B and PS E).

Capsular polysaccharides are a major virulence factor of *B. fragilis* and play an essential role in abscess formation. *B. fragilis* produces as many as nine capsular polysaccharides [31], and the seven of them phase vary via the inversion of promoters locate upstream the PS loci [32]. Of these, abscess forming capsular polysaccharides are only two types of capsular polysaccharides, PS A and PS B, in strain NCTC9343 [14], [15]. The remaining seven capsule types are not essentially involved in abscess formation. Interestingly, the expression of these PS loci in strain YCH46 is regulated by PhoB: PhoB is positively associated with PS E expression but negatively with PS B expression. These regulatory activities might be indirect since PhoB did not bind these PS promoters in the EMSA assay (data not shown). However, as reported by Chatzidaki-Livanis *et al.*, the transcription of each of the phase variable PS loci is not simply dependent on the promoter orientations but regulated in a complex manner, in which the UpxZ proteins that encoded by the second gene of each of the phase variable PS loci inhibit the synthesis of heterologous capsular polysaccharides [33]. It still remains the possibility that the *B. fragilis* PhoB bind to the PS promoters other than those of PS B and PS E, affecting the expression levels of these PS loci. Although *phoB* deletion reduced the transcriptional level of the PS A locus, the role of PhoB on abscessogenic PS A production is likely to be weak since the difference in the transcriptional levels of many genes in the PS A locus was small between wild type and Δ*phoB* mutant strains (Tables S3 to S6). Due to the heterogeneity of PS loci among *B. fragilis* as strains NCTC9343 and YCH46 share only the PS B locus [34], it is difficult to predict the physiological significance of PhoB regulation on polysaccharide biosynthesis in strain YCH46. Although the dose-dependency should be observed to compare the abscessogenic potential between the strains, our results in the mouse peritoneal abscess model indicate that these PhoB-regulated polysaccharides in strain YCH46 are not essential for abscess formation. Rather, these polysaccharides (PS B and PS E) and other Pho regulon genes are probably involved in survival of *B. fragilis* at extraintestinal sites such as the peritoneal cavity and the interior of abscesses.

PhoB has been associated with virulence in several pathogenic bacteria [3]. In many cases, the role of PhoB on virulence has been evaluated by the use of a constitutively active model obtained from mutation of the Pst system, which negatively regulates the Pho regulon, regardless of Pi concentration. In this model, PhoB activation reduced toxin production and pillus formation in *Vibrio cholerae* [35], and suppressed the growth of avian pathogenic *E. coli* in the intestinal tract [36]. However, Pho regulon genes have also been induced during infection of models with *Yersinia pestis*, *Erwinia chrysanthemi*, *Listeria monocytogenes* and *Mycobacterium tuberculosis* [4]–[8]. These findings indicate that the Pho regulon may work at particular stages of the infection process and that timely activation of this regulon is essential for adaptation. Therefore, constitutively active PhoB might interfere with the infection process.

In this study, it was concluded that PhoB does not directly affect the abscess formation of *B. fragilis* when virulence was assessed using a mouse model. The results also showed that the inflammatory cell infiltrations into peritoneal cavity by wild and Δ*phoB B. fragilis* strains were similar at an early stage (3 days) of infection. In addition, the viable cell number/abscess of both strains was similar when each strain was individually inoculated into peritoneal cavity (Table 4). On the other hand, the *phoB* deletion resulted in the faster clearance of the cells from abscess than wild strain in the mixed inoculation experiments, while the number of *phoB* mutant was similar to that of wild type strain in the GAM broth or mouse intestinal tract. However, it is possible that the mutant could not compete with the wild type for intestinal colonization under special conditions such as nutrient restriction and mucosal inflammation. These results indicate that PhoB regulates the stress adaptation of *B. fragilis* and contributes to survival of this anaerobe inside peritoneal abscesses. It is also possible that the increase of wild type to *phoB* mutant ratios in the abscess might only represent the difference in their growth rates. The monitoring of growth status of both strains and Pi-levels in the peritoneal abscess is necessary in the future study to determine the role of the PhoB in *B. fragilis.*

*Bacteroides* species are the causative agents of post-operative abdominal infections. Oral Pi administration prevents translocation of *P. aeruginosa* after surgical operations [11]. Signal transduction via the PhoRB system is a potential target for control of intra-abdominal infection by *B. fragilis*. The results presented here provide information that may be used to develop novel strategies for the handling of intra-abdominal infections to improve the recovery.

## Supporting Information

Table S1 **PCR primers used in this study.**
(XLSX)

Table S2 **Two-component signal transduction systems (TCS) in** ***B. fragilis*** **strain YCH46.**
(XLSX)

Table S3 **The wid-type genes whose expressions were induced >4-fold by Pi starvation.**
(XLSX)

Table S4 **The wid-type genes whose expressions were repressed >4-fold by Pi starvation.**
(XLSX)

Table S5 **The** ***phoB*** **mutant genes whose expressions were induced >4-fold by Pi starvation.**
(XLSX)

Table S6 **The** ***phoB*** **mutant genes whose expressions were repressed >4-fold by Pi starvation.**
(XLSX)
